# The role of national hospital associations in health system governance before and during the COVID-19 pandemic: Findings from an exploratory online survey

**DOI:** 10.1016/j.hpopen.2022.100077

**Published:** 2022-09-09

**Authors:** Bruno Meessen, Sara Perazzi

**Affiliations:** a Department of Health Systems Governance and Financing, World Health Organization, Geneva, Switzerland; b International Hospital Federation, Geneva, Switzerland

**Keywords:** Health system governance, Hospital, Policy, Private sector, Nonprofit

## Abstract

**Objective:**

Building on the premise that health authorities should govern their health systems in partnership with the full community of stakeholders, we document the contribution of national hospital associations to health policy processes, before and during the COVID-19 crisis.

**Methods:**

This research followed a rapid cross-sectional comparative design. Data were collected through an online survey targeting hospital associations. Eighteen of them shared information on their institutional profile, their areas of activity, their position and participation as policy actors before and during the COVID-19 crisis, the barriers and enablers affecting their participation and the impact of the crisis on their own financial situation.

**Findings:**

We have documented a spectrum of situations both for national policy platforms and hospital associations. In some countries, there is the ideal match of well-established associations and national participatory health policy platforms. In others, hospital associations have modest staffing and may struggle to get access to policy platforms of importance. Being a well-established and respected contributor seems to have been an enabling factor for the contribution of the hospital associations to the COVID-19 response. For most associations, the crisis has led to an increased effort to be present in the policy arena; an issue they follow closely is the negative impact of the lockdown on the hospitals’ revenue.

**Conclusion:**

The growing pluralism characterizing our societies calls for the establishment of health policy platforms allowing for broader participation. Encouraging hospitals to set up their association for the latter to represent them in decision processes could be one of the components of the rebuilding of national health systems post pandemic.

## Highlights

• Growing pluralism characterizing our societies calls for more attention to the contribution of different health system stakeholders to policy processes.
• This study documents the roles of eighteen national hospital associations before and during the COVID-19 pandemic.
National authorities should grant a role to national hospital associations in the rebuilding of health systems post pandemic.

## 1 Introduction

Liberalization, marketization, and citizen emancipation are accelerating the transformation of our societies. In the health sector, just like in other sectors, new modalities to organize the social order are emerging. Over the last decades, many countries have moved away from centralized governance model (where power and policy instruments are mainly in the hands of the Ministry of Health) to models allowing for a shared governance approach valuing the voices and contributions of the numerous stakeholders of the health system [1], [2].

At the World Health Organization (WHO) and International Hospital Federation (IHF), we believe that platforms allowing for consultation, dialogue and participation are particularly key when health systems are composed of a wide variety of independent actors [3], [4]. Seeing intrinsic value in participation and supporting participatory governance at country level (versus alternatives such as autocratic governance, corporatist governance, or in low-income countries, governance dominated by external actors) do not evacuate the need for critical attention. Participatory governance models raise important questions [4], including whom represents the various actors on the policy platforms and how these representative bodies emerge and operate.

In this respect, hospitals are an interesting class of actors. In a growing number of countries, hospitals – even public ones – hold substantial autonomy. Being increasingly responsible for their own economic viability, hospitals take a variety of initiatives favorable to their activity and economic development. Setting up or joining a hospital association is one of them.

There is limited knowledge today on the contribution of hospital associations, or more broadly national health service provider organizations (NHSPO), to national health policy processes. The hypothesis behind this paper is that there is room for a dedicated research agenda. For the structuration of the latter, the concept of ‘meta-organization’ proposed by Ahrne and Brunsson might be helpful [5]. Most NHSPO indeed match the three definitional characteristics of meta-organization: (i) they are organizations, i.e., ‘decided social orders’; (ii) they are associations and their members form the center of authority; (iii) their members are themselves organizations (and not individuals). Questions abound: what is the profile of NHSPO as meta-organizations; what are the factors supporting their emergence; which services do they provide to their hospital members; how do they contribute to health policy; are there pitfalls to worry about?

The COVID-19 pandemic has brought a lot of attention on health system governance processes [6], [7]. The crisis has been testing the resilience of our societies at many levels, including the capacity of governance systems to rapidly manage the consequences of the crisis and put short term and long-term solutions into place. In this study, we use the COVID crisis to explore the contribution of NHSPO to policy processes. The COVID crisis has constituted an unprecedented economic shock for the hospital sector. Because of the surge in operational costs on one hand and of the major reduction of revenue caused by the lockdowns on the other hand, autonomous hospitals have been under unprecedented financial pressure [8]. This financial crisis will have deep and long-lasting impact on the economic situation of many hospitals. NHSPO have not remained passive.

This study belongs in a program of work launched by WHO and IHF to document both the economic problems experienced by hospitals because of the COVID-19 crisis and the policy measures taken to relieve the sector. In line with normative premise stated above [health authorities should govern their health systems in partnership with the full community of stakeholders and their representatives] [4], [9], we first surveyed national hospital associations, with an online survey focusing on the role played by national hospital associations before and during the COVID crisis. A better understanding of their role is key for the full recognition of their contribution to well performing and resilient health systems.

After having presented the methodology of the online survey, we report the data from the 18 national health service providers organizations (NHSPO) which have participated to the survey. Our discussion section is organized around two main considerations. Firstly, we discuss the meaning of our findings for the documented NHSPO, which are mainly in high-income countries (HIC). Secondly, we argue that this learning agenda is also relevant for countries where policy platforms for shared governance and representative bodies such as hospital associations are under-developed and not yet institutionalized [3]. We identify several tracks for further research.

## 2 Methods

The IHF-WHO program of work on hospitals in times of COVID-19 is designed as an iterative one. The first step was to engage with national health service providers organizations (NHSPO), which are key holders of knowledge on the hospital sector in their respective country and close observers of the implication of any external shock or policy measures on their members. Building a strong relationship at the early stage was also a prerequisite for our second survey which will target their hospital members. We took the opportunity of our first interaction with the NHSPO to develop a better understanding of their role in national policy processes, before and during the COVID-19 crisis.

This research followed a rapid cross-sectional comparative design. Data were collected through an online survey targeting hospital associations. The survey questionnaire was developed with four main objectives in mind: (1) to describe NHSPO [a basic information missing so far]; (2) to document their areas of activities before and during the COVID-19 crisis; (3) to collect information on policy measures taken at national or sub-national level to relieve the hospital sector from the economic consequences of the COVID-19 crisis; (4) to give NHSPO the opportunity to report on the impact of the crisis on their own situation. The structure of the questionnaire loosely follows the so-called policy triangle, which focuses on content of a policy, by organizing the analysis of it in the triangle of context, actors and processes [10]. One question, on policy involvement, follows the policy process heuristic framework [11]. The questionnaire was developed with a concern to allow for heterogeneity across contexts, NHSPO, areas of activity, and participation to policy processes and the COVID response.

The questionnaire was developed by the first author and after test and review by different experts of a steering group (see acknowledgement section), was slightly revised. An invitation to participate to the survey and an accompanying guidance note were sent to national hospital association members of the IHF and of the European Union of Private Hospitals, respectively 27 and 13 associations. For the online administration (in English only), we used LimeSurvey, as hosted on the WHO server. This survey was exempted from ethical review, as respondents were invited to report the views and opinion of their associations, not their personal one. In the guidance note, it was acknowledged that completing the survey would probably require to consult several persons in the organization and therefore recommended to share the questionnaire file with all the relevant people in order to collect full insights and then entrust the survey completion to one designated person.

An informed consent was signed electronically by each participating NHSPO. Our data analysis was very straightforward. Given that our sample is small, we limited ourselves to simple descriptive analysis.

In this paper, we report on: (1) the key characteristics of the NHSPO; (2) their areas of activity, their position and participation as contributor in the policy arena before and during the COVID-19 crisis, including an investigation of barriers and enablers affecting their participation to the response; (3) the impact of the crisis on the financial situation of the NHSPO.

As a validation step, our data and conclusions were presented to participating NHSPO in a webinar. They also got the opportunity to read this paper and to suggest revisions. Along our iterative approach to this new research program, our results should be more seen as emerging hypotheses requiring more investigation in the future than robust findings.

## 3 Results

### 3.1 Pre-COVID

Eighteen NHSPO participated to the survey (Table 1). Most of them are European associations. Ten NHSPO represent private hospitals only (here after reported as NHSPO-Pr); others are either focusing on public hospitals or have a mixed composition (here after reported as NHSPO-Mx). One association, Unicancer, has an exclusive membership of hospitals specialized in cancer treatment. The German Hospital Federation (GHF) has a membership of associations. HealthCare*CAN* practices a system of different membership rights to accommodate the variety of organizations interested in its activities.

**Table 1:** Description of the NHSPO.

	**Country**	**Members**	**Number of Full-Time Equivalent (2019)**	**Year of foundation**
Association of Private Hospitals in Austria	Austria	78	2	1953
SANTHEA	Belgium (Fr)	37	26.6	1963
Brazilian Hospitals Federation	Brazil	4,196	8	1966
HealthCare*CAN*	Canada	56, with different profiles and rights	21	1931
French Hospital Federation	France	4,800	34.4	1924
Unicancer (previously *Fédération Nationale des Centres de Lutte Contre Le Cancer*)	France	20	197	1964
German Hospital Federation (GHF)	Germany	16 state hospital associations and 12 national associations	100	1949
Italian Association of Private Hospitals	Italy	520	72	1966
Private Hospitals Association Jordan	Jordan	59	4	1984
*Syndicat Patronal Monégasque des Etablissements du secteur sanitaire et social*	Monaco	2	0	1995
Polish Association of Private Hospitals	Poland	153	2	2002
Portuguese Association of Private Hospitals	Portugal	62	6	1971
Portuguese Association for Hospital Development	Portugal	21	4	2002
*Patronatul Furnizorilor de Servicii Medicale Private din Romania - PALMED*	Romania	25	6	2007
Catalan Hospital, Health and Social Services Association - Unió Catalana d'Hospitals	Spain (Catalonia)	114	16	1975
Spanish Private Health Alliance	Spain	1,300	6	2016
Swiss Private Hospitals	Switzerland	103	1	1980
American Hospital Association (AHA)	USA	4,188	481	1898

The 18 associations have very different sizes. Biggest associations are based in big countries and may have more than 4,000 hospital members. Some have a lot of human resources, whilst others rely on a few individuals. The American Hospital Association (AHA), has more than one hundred years of existence. A few associations are quite recent.

One of our expectations was that NHSPO operate in very different environments for the institutionalization of national policy platforms. Each respondent had to rate the status of the existing policy platforms in their country (defined as “*committees, commissions, formal bodies, working groups where an organization like yours has the opportunity to engage with other stakeholders and the health authorities in particular”)*. They had to report whether they “strongly disagree”, “disagree”, “are not sure”, “agree” or “strongly agree” with seven different statements (Table 2). We have recoded these answers respectively as −2, −1, 0, 1 and 2. In Table 2, we report the average score for both types of NHSPO. Given the very small populations of the two groups, these statistics must be taken as exploratory only. There is a pattern, across statements, of more negative assessments by NHSPO representing private hospitals. In Portugal, a country for which we have data from both categories, the two associations agree only on statements 5 and 7; on all the others, the NHSPO-Pr is more critical than the NHSPO-Mx. Among the 18 associations, the GHF stands out, as it reports to operate in a context of well-established, well-organized and effective platforms mobilizing contributors with clear mandates (see, further our Box 1 which provides more information about this interesting case). Again for exploratory purposes only, we also looked at the correlation between the age of the association and the scoring on each attribute. If age matters, it seems limited to attributes 2 and 7.

**Table 2:** Status of policy platforms (18 NHSPO).

**Attribute**	**NHSPO-Mx (n = 8)** **Average score**	**NHSPO-Pr (n = 10)** **Average score**	**Correlation (*r*) with age of the association**
1. Policy platforms have been institutionalized for a while. They have clear and permanent mandates (e.g. established by decrees)	1.13	0.3	0.10
2. All relevant stakeholders are part of the policy platforms	0.88	−0.1	0.31
3. Participants to policy platforms have themselves clear mandates from well-identified constituencies	1	0.2	−0.04
4. The functioning of policy platforms is very well organized (e.g. agenda, minutes of meetings, decisions)	0.88	−0.1	−0.05
5. Stakeholders comply with decisions taken at policy platform meetings	0.75	0.6	0.03
6. Authorities rapidly set up new specific platforms (e.g. working groups) if it is appropriate	1.12	0.6	−0.17
7. There are mechanisms to share in a fluid way relevant knowledge among stakeholders	0.88	−0.1	0.42

Box 1 **The German Hospital Federation’s contribution to health policy processes during the COVID-19 crisis.**

**Agenda setting:** An important task of the GHF was, and still is, to create awareness of the financial and economic risk as well as of the administrative challenges threatening hospitals. GHF alerted policymakers to the financial risk coming along with the downscaling of activities (decrease in income) and with the new crisis-related measures to finance, for example, the newly installed isolation areas and intensive care beds or the increased costs for PPE (increase in expenditures). Different communication channels, for example press releases and media platforms but also direct contact via letters, e-mails and phone calls have been used. The concern was to ensure that the search for a suitable solutions (ex. financial rescue package for the economic stability of hospitals, suspension of certain complex administrative regulations, crisis-related flexibility on Nurse-Patient-Ratios, COVID-19-testings of hospital patients, as well as of hospital staff on a regularly basis at a cost covering level) was on the top of the political agenda.

**Policy formulation:** In the German legislative process on the national level, drafts of national laws and regulations are transferred to the relevant stakeholders to invite them to comment, as experts, on the solutions submitted. The GHF comments on every draft that may affect hospitals. Its experts comment on the suitability of every measure proposed and suggest adaptions and formulations in line with hospitals' interests. This procedure is also valid and used in times of crisis. However, during this crisis, the GHF has been consulted more often to give expertise on hospitals' actions and needs.

**Policy implementation:** After a national law or a regulation has entered into force, the GHF processes the content and measures in order to inform its members adequately and in a timely manner about the changes that will affect hospitals. This procedure is also valid in times of crisis. However, the amount and the frequency of information that the GHF processes for its members is much higher than before the crisis, as there are more measures introduced about which hospitals need to be informed. National laws sometimes set a general framework and under a logic of self-government system, healthcare organizations have to decide on many binding details within this framework. This procedure is also valid and used in times of crisis.

**Monitoring and Evaluation:** With the “financial rescue package for the economic stability of hospitals” a monitoring committee on the short-term/ middle-term/ long-term suitability of the measures was implemented. Representatives of the GHF were nominated to evaluate and monitor the situation and to suggest adaptions within this committee to be convened by the Ministry of Health.

To understand the domain of activity of the associations, we have asked them to score a set of possible activities (‘not an activity for us’, ‘it happens (rarely)’, ‘one of our activities’, ‘strategic activity’) as they were carried out in 2018–2019 (before COVID-19). We believe that ‘strategic activity’ (last column) gives a good sense of the focus of most associations: it is on following and contributing to health policy (items 1–5). There seems to be some policy activities easier than others (sitting on a formal policy forum is probably easier than pro-actively proposing policy measures – the latter requires advanced policy formulation capacity). A pattern also emerges for communication and knowledge management (items 11–19): communication activities (items 11–12) are a strategic activity or ‘one activity’ for a strong majority of associations; activities mobilizing collective intelligence of hospital members (items 13, 17) and analytical work (items 18–19) seem also privileged; conversely most NHSPO have modest engagement for activities requiring to ‘know more’ than hospital staff (items 14–16). So there seems to be a general pattern of specialization in intelligence and knowledge management activities which are synergetic with policy engagement (e.g. maintaining a website, being present in media, carrying out analyses). Before COVID-19, the involvement in economic negotiation on behalf of hospital members was limited (items 6–10). What is done by most, again, is what is mainly synergetic with the policy engagement (negotiation with public funders). Few associations try to market their own expertise (items 20–21). The ‘other’ question brought to light the international role played by several associations (to represent the MoH on international platforms, to promote members abroad for the niche of medical tourism). HealthCare*CAN* also flagged its contribution to the production of policy briefs, a knowledge management practice well-established in Canada. A few associations look more specialized than others – one can hypothesize that in some countries, some functions are already fulfilled by another body or entity. The association which reported the broadest set of strategic activities is the AHA. Its maturity and scope are also materialized in its investment in maintaining professional membership societies for functional leaders within hospitals (e.g., nursing executives, facility leaders, strategic planners).

### 3.2 During COVID-19

For all countries of the world, the COVID-19 pandemic was a major unexpected event. We have tried to assess to what extent the hospital associations have played a role in the response, especially in the development of the measures to mitigate the consequences upon hospitals as economic entities.

A first question focused on their involvement in the preparation the few weeks before the declaration of the first case in their country (emergency preparedness). In Germany, the first case came so early that no specific preparation was already in place. As for the other 17 NHSPO, three were rapidly involved – one of them (Jordan) because its chair was already present in all the right national committees (also because of his personal profile). Seven associations requested to be involved; among those, three have seen their request rejected, all of them representing private hospitals; one of those which reported to have managed to be involved reported that it took some time (because authorities were not well organized) and was partial only (some of their requests for some platforms were rejected). The seven other associations were not involved, sometimes for logical reasons (for instance, because the preparation was handled by another level in a federal country, because the association gathers cancer treatment facilities).

Participating to policy platforms is certainly an important function for most NHSPO of our sample, but one can also see in Table 3 that all of them have some activities targeting their members. We therefore also asked them to characterize the emergency preparedness support that they provided to their members before the declaration of the first case. Eight associations reported that they did not bring value to their members (e.g. emergency preparedness is the responsibility of a specific authority), seven consider that they brought some value but acknowledged that it was probably minor, three estimate that they played an important role for the preparation. The main contribution was to support the collection of information (e.g. availability of beds), pro-active engagement with members to check their needs (e.g. in terms of personal protective equipment, PPE), relay issues to the national authorities (e.g. shortfall of PPE). One federation set up an ‘Infocovid weekly newsletter’; another one reformatted a forthcoming face-to-face training into an online version and developed its own plan for the times to come.

**Table 3:** Domains of activity of the 18 NHSPO before COVID-19.

	**Not an activity for us**	**It happens (rarely)**	**One of our activities**	**Strategic activity**
1.Contribute to the development of the national/regional hospital strategy	2	4	4	8
2.Represent members in formal policy forums	1	2	4	11
3.Monitor regulations and policies affecting members	2	1	4	11
4.React to policy measures harming members	0	2	6	10
5. Pro-actively propose policy measures benefiting members	0	4	7	7
6.Support members in negotiation with public funders	3	3	6	6
7.Support members in negotiation with private funders (e.g. private insurance)	11	0	6	1
8.Support members in negotiation with suppliers	11	3	2	2
9.Support members in negotiation with workforce (doctors, unions…)	9	1	5	3
10.Support members to find the best fit in the health system (e.g. relationship with first line services)	7	6	5	0
11.Represent members in general media (TV, radio, press)	1	1	9	7
12.Share information via online channels (newsletter, website)	1	0	10	7
13.Facilitate peer-to-peer learning among members (workshops, online discussion group, webinars)	3	3	6	6
14.Provide coaching to hospital managers	9	3	5	1
15. Organize training for members or their staff	6	4	4	4
16. (Co–)produce guidelines	6	6	3	3
17. Organize conferences for the hospital sector	1	3	8	6
18. Contribute to studies or collection of data	1	2	11	4
19. Carry out analyses and produce reports	1	2	8	7
20. Deliver services (e.g. consultancies)	5	4	7	2
21. (Co–)develop products or solutions for members (e.g. software)	10	5	3	0
22. Other (to specify in the next question)	12	0	3	3

Our next question was about the NHSPO’s contribution during the next stage of the crisis: the surge of COVID-19 admissions; in all countries of the world, hospitals have been at the frontline. We proposed a list of 14 possible areas of action, and we asked the NHSPO to rate their contribution (Table 4). One sees a quite spread distribution of involvement, with a heavy presence in information matters, consistent with the fact that many associations were already knowledge management and intelligence hubs before the crisis. One can also look at the data per NHSPO; three patterns emerge. Several NHSPO had no or light involvement across all the items – the job has been done by other actors in the health system. Some NHSPO had a limited involvement but very focused, with sometimes leadership – for instance, Unicancer which was already in charge of the relationship with suppliers before the outbreak was in the frontline to procure COVID items. A few had a very active role. For instance, the Brazilian Hospitals Federation and the AHA have reported a leadership role on eight and nine areas of action respectively, and a presence across fourteen and thirteen areas respectively – maybe to compensate the uneven-ness of the response by other policy actors.

**Table 4:** Contribution of the 18 NHSPO during the surge of COVID-19 admissions.

	No involvement	Light involvement	Significant involvement	Active leadership
Information to the public	2	3	6	5
Information to hospital staff	3	1	4	8
Procurement of COVID supplies	5	2	5	5
Procurement of non-COVID items	8	2	4	3
Protocols for screening, tracing or referral at health system level	6	3	3	3
Protocols or electronic solutions for COVID data management	9	3	1	3
Collaboration with other health service providers for the response	4	3	6	4
COVID treatment protocols at hospital level	7	5	3	1
Safety measures for hospital staff and patients	5	5	4	3
General human resource management	5	6	6	0
Safeguarding the delivery of non-COVID essential services	5	3	3	4
Adjustment to non-COVID non-priority services (e.g. elective surgery)	4	5	3	2
General administrative and financial management	5	3	3	4
Reorganisation of ancillary services (e.g. catering)	14	1	1	0

No segment of our societies has been safe from the COVID-19 crisis. We asked participants to assess how the crisis has affected their own operations by using the domains of activity previously used for the period before COVID-19 (see Table 5). One clearly sees that all NHSPO maintained or, more often, accentuated their presence on policy platforms (items 1–5) – this is consistent with the fact that in 2020, all countries had to fast track health policy measures to address the crisis, with hospitals playing a pivotal role in the clinical management of COVID patients. One will notice that the vast majority of NHSPO had to increase their attention to defending their members (item 4). Several also developed a new presence in transactional relationships (items 6–10) – supply has clearly been a high issue in 2020. As for knowledge management and intelligence, one sees, without surprise, a shift away from face-to-face conferences (item 17) to formats compatible with social distancing rules and the need to communicate a lot of information in a swift manner (items 11–13).

**Table 5:** Domains of activity of the 18 NHSPO during COVID (2020).

	**Increase**	**Same**	**Decrease**
1.Contribute to the development of the national/regional hospital strategy	9	9	0
2.Represent members in formal policy forums	11	7	0
3.Monitor regulations and policies affecting members	7	11	0
4.React to policy measures harming members	14	4	0
5. Pro-actively propose policy measures benefiting members	12	6	0
6.Support members in negotiation with public funders	7	11	0
7.Support members in negotiation with private funders (e.g. private insurance)	4	14	0
8.Support members in negotiation with suppliers	7	11	0
9.Support members in negotiation with workforce (doctors, unions…)	4	14	0
10.Support members to find the best fit in the health system (e.g. relationship with first line services)	4	13	1
11.Represent members in general media (TV, radio, press)	12	5	1
12.Share information via online channels (newsletter, website)	15	2	1
13.Facilitate peer-to-peer learning among members (workshops, online discussion group, webinars)	9	7	2
14.Provide coaching to hospital managers	5	13	0
15. Organize training for members or their staff	2	13	3
16. (Co-)produce guidelines	5	11	2
17. Organize conferences for the hospital sector	4	6	8
18. Contribute to studies or collection of data	9	7	2
20. Deliver services (e.g. consultancies)	5	10	3
21. (Co-)develop products or solutions for members (e.g. software)	1	15	2
22. Other (to specify in the next question)	2	15	1

According to the heuristic policy process framework [11], a policy process can be divided into four stages. *Agenda setting* refers to expressing the need and creating pressure for getting 'something done'. *Policy formulation* refers to designing the policy, defining its content, allocating financial resources and issuing official texts. *Implementation* refers to communicating and explaining the measure, organizing the training, ensuring availability of required capacities, adapting and enforcing the routines. *Monitoring and evaluation* refer to observing the uptake, execution and effects. We have asked the NHSPO whether they have participated to any of these four stages as for the COVID-19 policy measures taken by health authorities in favor of hospitals. Eleven associations reported a contribution as for agenda setting, twelve for policy formulation, thirteen for implementation and eleven for monitoring or evaluation. All the associations reported an involvement in at least one of the four stages. The AHA, the GHF, the French Hospital Federation and SANTHEA in Belgium have managed to contribute at several moments of the policy process. For associations with smaller human resources, contributions have been more limited. The questionnaire encouraged NHSPO to provide, through comment open-ended questions, more detailed information on their specific experience. As an illustration, Box 1 details how the GHF has contributed across the different stages of the policy process. From a more prescriptive perspective, it gives a sense of the quite advanced possible contribution of a NHSPO when it operates in the context of a well-established participatory health system governance set-up.

Sometimes, contributing to policy making is about arguing against inappropriate policies or at least actively raising awareness about their consequences. To the question whether they were aware of policy measure taken by national or local authorities which had harmed or could have harmed the performance or the financial situation of hospitals of their network, most associations stressed the very negative economic impact of the lockdown on the normal activity of their members. Associations have been very active on this agenda.

We invited respondents to flag any contextual factors which may have affected the COVID response and more particularly their participation to it. Several respondents stressed that the decentralized nature of their health system has been a constraint both for the response and for their participation in it. For instance, in Belgium, the health policy area falls under the jurisdiction of different national and subnational governments; this has complicated quite a lot the communication between all relevant authorities and required SANTHEA to duplicate its engagement effort with all relevant authorities. In some countries, the fact that private hospitals were not as well integrated into the health system as public ones has also been a constraint for the NHSPO. A NHSPO with membership from the private sector offered twice its assistance at different stages of the crisis and its offer was each time rejected.

A couple of questions focused on possible factors which enabled or disabled the contribution of the NHSPO to the COVID-19 response. The quality of relationships with authorities and members and sitting at the right policy platforms before the crisis were key enablers. NHSPO-Pr reported less enablers and more barriers than NHSPO-Mx – the main one being not to be seen as priority contributors.

A last set of (facultative) questions related to the economic situation of the NHSPO itself. Eight associations reported that the crisis had a negative or very negative impact on their revenue. Six reported that it was neutral. Only one reported a positive impact. We asked NHSPO whether they had been eligible to any scheme put in place to protect business and organizations from the economic consequences of COVID. Only 3 reported positively. Most were pessimistic on the prospect of being assisted. We put forward a few scenarios and asked NHSPO to give them some probability. Five among 12 respondents gave a high or very high probability score to the scenario “Managers will have to take difficult decisions (cost reduction, firing of staff, sale of assets, renegotiation of contracts, activity scale-down…)”. A young federation reported a high probability to the scenario “Our existence will be in jeopardy”. Conversely, well- and long-established associations gave a very low probability to that fate.

## 4 Discussion and Conclusion

To our knowledge, this is the first cross-country documentation of the contribution of hospital associations to national health policy. Our research has the obvious limitations of an online survey covering a small population and without triangulation, yet, we believe it provides an interesting first iteration for this future research agenda.

We have documented a spectrum of situations both as for national health policy platforms and hospital associations. In some countries, like Germany, there is the ideal match of well-established and well-staffed associations and a tradition of national participatory policy platforms. In others, hospital associations are younger, have modest staffing and activity portfolios and may still struggle to get access to policy platforms of importance.

Surveyed associations have reported variable contribution during the emergency preparedness and response stages. There are of course idiosyncratic factors (e.g. the federal nature of a health system), but it seems that several well-established and respected NHSPO were able to contribute on many aspects. For most associations, the crisis has led to an increased effort to be present in the policy arena – a major area of engagement has been and is still the negative consequences of the lockdown on the hospitals’ revenue.

Our research gives some directions to be considered by national health authorities. Health system governance arrangements must be in step with the transformations of the health sector landscape underway. Over the last decades, in many countries, public hospitals have been granted greater autonomy. This extra decision space is precious given the fact that, in their endeavor of optimizing patients’ pathways for best health outcomes, hospitals must constantly recombine a wide spectrum of individual expertise, team capacity, therapeutic and technological innovations and infrastructure. The importance of allowing such agility has been demonstrated with the COVID crisis. As the old administrative hierarchy lines are vanishing, hospitals need new mechanisms to organize their interaction with national health authorities but also with other stakeholders. This necessity of formal mechanisms is even truer for private hospitals. Many low- and middle-income countries (LMICs) are lagging in terms of both regulatory frameworks and policy platforms for this category of providers [3], [12]. The emergence of NHSPO and their participation in a transparent manner to public policy processes is part of the healthy development of a pluralistic health system [13] and is certainly preferable to clientelism and personalized networks.

Could this emergence be supported? Surely, no one will organize the hospital industry against its own will: it is up to hospitals to take the initiative, establish their association, appoint their representatives and inform national authorities about their willingness to contribute to the development of the national health system. As our study as shown, each association will also develop the original portfolio of activities which best fits the needs of its members. But all their efforts can be welcomed and encouraged by national health authorities. The increasing pluralistic composition of the health sector calls for new attitudes and practice in terms of leadership [14]. As stewards of the health system, ministries of health should acknowledge the role played by all categories of health service providers and establish an environment favorable to their consultation and participation [4]. Ideally, this effort should be part of a broader commitment to enhancing social participation for more effective and equitable health policies, with sufficient investment into new competencies such as the ability to amplify the voices of those traditionally left behind, the capacity to manage conflicts of interest, and skills in dealing with potent interest groups [7].

One issue will be to establish, from day one, the collaboration with NHSPO at the right level. Whilst a hospital association is in its mandate when it defends the interests of its affiliates, efforts must be done to establish a culture of goodwill supportive to the strengthening of the whole health system, including primary health care. Our assessment is that global actors, like the WHO and the IHF, but also donors, could be helpful in the consolidation of such hospital associations supportive to larger societal goals. In many countries, the COVID-19 crisis has been a real test on the capacity of health actors to work together towards a common goal: where trust has been built, there is a window of opportunity to seize.

These normative orientations (to support the emergence and contribution of NHSPO in health system governance) would certainly benefit from more evidence. Our work, though just exploratory, gives some possible directions to the research community.

In terms of research design, the fact that they are not that many national hospital associations worldwide suggests limited prospects for surveys and quantitative analysis. The most promising pathway is probably to embrace qualitative methods and produce case studies, including comparative ones, embedded in the analysis of their health sector and policy context.

As mentioned in the introduction, one source of inspiration for this case study work might be the literature related to so-called ‘meta-organizations’ [5]. One of the interesting insights from the meta-organization literature is the study of factors favorable to their emergence [15]. To study the emergence and development of long-established NHSPO in some HICs, researchers could combine the insights of the meta-organization literature with the methodological approaches being developed by organizational historians [16]. In LMICs, there are certainly some interesting cases of recent emergence; archives of the NHSPO will be less rich, but researchers should certainly not give up historical analyses. In his study of the contribution of NHSPO to the politics of health care in Turkey, Yilmaz shows how their emergence fit within the relational nexus between the State and business organizations and the broader history of development of the country. One can expect that studies of recent emergence will bring a broad set of actors and influences (e.g., the different development paradigms which have prevailed after the second World War). Doing research in countries where NHSPO are still missing would also be relevant. This absence is intriguing, especially in countries with a dynamic private hospital sector. What are the factors inhibiting the emergence of meta-organizations to defend its interests? Is it a deficit of space for participation… or a surplus of informal channels for clientelism? The COVID-19 crisis has revealed that this question matters from a policy perspective [17]: in several countries, in the early days of the response, national authorities lacked well-identified counterparts to discuss and negotiate the involvement of private hospitals in the response.

This suggests another research agenda, with a more normative perspective: what is the right place for NHSPO in health system governance? An interesting body of literature will be the one relating to health system governance. There has been an ongoing shift in this field: analysts have moved away from an understanding of governance as a function to be fulfilled by governments (and ministries of health in particular) to a recognition that governance is fundamentally about the organization by human beings of their collective action [9], [18], [19]. With such a framing, one can see that setting up and maintaining a hospital association allows hospitals to pool resources to address some common problems related to their own functioning (e.g. organize workshops to share experience and practice on how to operate), build a common identity (thanks to the experience of a same condition in front of common external constraints) and organize their action towards external actors (e.g. lobby national health authorities).

For evidence-informed guidance, it could be beneficial that political and health system scientists more systematically embrace a pluralistic view of the stakeholders contributing to health policy processes and sector reforms: unions, political parties, professional groups, patient associations, lobbies, national NGOs… This is particularly needed in LMICs where much of the attention still goes to the ‘donor-government duel/duet’. There is an emerging literature looking at the role of professional groups [20], [21], but very little is still known about the participation of NHSPO in policy processes [22], [23]. Importantly, granting legitimacy to NHSPO – as we did in this paper – does not entail any endorsement of their specific policy agenda in a specific country in a specific point of time. Researchers should keep in mind that there are possible issues with the alignment of NHSPO action with desirable goals such as Universal Health Coverage and ‘Leave no one behind’ – e.g. defense of high payment rates, promotion of a hospital-centric view of the health system against the need in many countries, including HICs, to invest much more in promotional and preventive services. Henceforth, independent scientific scrutiny of their action will be crucial. This is a third research agenda, where critical perspective on health system development and performance and methods such as stakeholder analysis will be key.

Societies and health systems are under transformation. COVID-19 has shown the importance to have platforms for different stakeholders to bring their issues, share their views and analyses, inform reforms and develop ownership of new health policies. Our study has illustrated the contribution of NHSPO in eighteen countries. We believe that NHSPO have a role to play, across the country economic spectrum. Encouraging their establishment, inviting them to national policy platforms and documenting their effectiveness should be fully part of the rebuilding of our health systems post pandemic.

### CRediT authorship contribution statement

**Bruno Meessen:** Conceptualization, Methodology. **Sara Perazzi:** Conceptualization, Methodology, Project administration.

## Declaration of Competing Interest

The authors declare the following financial interests/personal relationships which may be considered as potential competing interests: The International Hospital Federation (IHF) is a global not-for-profit, non-governmental membership organisation. The IHF represents 100 different organisations across the global, including NHSPO.
